# Acute Ischemic Stroke: Management Approach

**DOI:** 10.5005/jp-journals-10071-23192

**Published:** 2019-06

**Authors:** Chandril Chugh

**Affiliations:** Department of Interventional Neurology, MAX Saket Hospital, New Delhi, India

## Abstract

**How to cite this article:** Chugh C. Acute Ischemic Stroke: Management Approach. Indian J Crit Care Med 2019;23(Suppl 2):S140–S146.

## INTRODUCTION

Stroke is defined by the World Health Organization as a clinical syndrome consisting of rapidly developing clinical signs of focal (or global in case of coma) disturbance of cerebral function lasting more than 24 hours or leading to death with no apparent cause other than a vascular origin.^1^ Stroke is classified broadly into three categories; ischemic stroke, hemorrhagic stroke and subarachnoid hemorrhage. Ischemic stroke occurs due to blockage of blood vessel which limits the blood supply to the brain whereas hemorrhagic stroke occurs due to rupture of blood vessel leading spillage of blood in the intracranial cavity.^2^ Depending on the site of blood spillage the hemorrhagic stroke could be classified as intracerebral hemorrhage or subarachnoid hemorrhage. Approximately 60–80% of all strokes is ischemic. This article is dedicated to acute ischemic strokes and its management.

## EPIDEMIOLOGY

Stroke is the second most common cause of mortality and third most common cause of disability worldwide.^3^ Globally, 68% of all strokes are ischemic and 32% are hemorrhagic.^3^ Numbers from the USA differ a little with 87% of all strokes being ischemic, 10% hemorrhagic and about 3% being subarachnoid hemorrhage.^4,5^ Data regarding prevalence of stroke in India is lacking however, it can extrapolated from the data available from the West. In a study done by Banerjee et al. in 2001 crude prevalence rate of stroke in India was 147/100,000 and the annual incidence rate was 36/100,000. Women had substantially higher age-adjusted prevalence rate (564/100,000 for women vs 196/100,000 for men) and incidence rate (204/100,000 for women vs 36/100,000 for men).^6,7^ Overall prevalence of stroke ranges from 147–922/100,000 in various studies.^8,9^

India has the highest burden of acute coronary syndrome (ACS) in the world and the three most common risk factors for ACS are smoking (40%), high blood pressure (38%), and diabetes (30%).^10^ Taking account of the above mentioned data and considering the fact that stroke shares common risk factors with ACS, we can safely assume that India has a very high incidence of stroke as well.

### Ischemic Stroke Classification

According to the multicenter Trial of Acute Stroke Treatment (TOAST) there are three kinds of ischemic stroke:^11^

1. Large vessel stroke
2. Small vessel stroke or Lacunar stroke
3. Cardioembolic stroke

Large artery strokes could be due to thrombotic or embolic occlusion of the major arteries of the brain like the internal carotid artery, middle cerebral artery, anterior cerebral artery or the vertebrobasilar system. Lacunar strokes are more often due to involvement of smaller or perforating blood vessels supplying the deeper structures of the brain.

### Risk Factors: Non Modifiable and Modifiable

#### Non Modifiable Risk Factors Include

- Age
- Race
- Sex
- Ethnicity
- History of migraine headaches
- Fibromuscular dysplasia
- Heredity: Family history of stroke or transient ischemic attacks (TIAs)

### Modifiable Risk Factors Include

- Hypertension
- Diabetes mellitus
- Cardiac disease (see below)
- High cholesterol
- Previous stroke
- Carotid stenosis
- Hyperhomocystinemia
- Lifestyle issues: Excessive alcohol intake, tobacco use, illicit drug use, physical inactivity
- Obesity
- Oral contraceptive use/postmenopausal hormone use

Majority of the ischemic strokes seen in patients with cardiovascular disease are embolic. Embolic strokes may arise directly from the heart or the aorta. Following is the list of conditions that carry a high risk for embolic strokes.^12,13.^

#### Arrhythmias

- Atrial fibrillation and paroxysmal atrial fibrillation
- Sick sinus syndrome
- Sustained atrial flutter

#### Valvular Heart Disease

- Rheumatic mitral or aortic valve disease
- Bioprosthetic and mechanical heart valves
- Fibrous nonbacterial endocarditis (ie, Libman-Sacks endocarditis), antiphospholipid syndrome, and cancer (marantic endocarditis), systemic lupus erythematosus
- Infective endocarditis

#### Thrombus and Structural Lesion

- Papillary fibroelastoma
- Left atrial myxoma
- Atrial or ventricular thrombus

#### Structural Heart Disease

- Recent myocardial infarction (within one month)
- Chronic myocardial infarction together with ejection fraction <28%
- Congestive heart failure with ejection fraction <30%
- Dilated cardiomyopathy

#### Surgical

- Coronary artery bypass graft (CABG) surgery
- Conditions which have been associated with embolic stroke but lack definitive evidence are mitral annular calcification, patent foramen ovale, left atrial smoke on echocardiogram, atrial septal aneurysm, left ventricular aneurysm and aortic arch atheroma.
- Genetic diseases, storage diseases, traumatic vascular diseases are well known causes of stroke which are beyond the scope of this chapter.

### Clinical Presentation

Clinical presentation of stroke depends on the area of the brain affected by occlusion of the arteries. AHA/ASA (American heart association/American stroke association) have popularised the FAST algorithm to diagnose stroke in the prehospital setting. FAST acronym stands for facial droop, arm weakness, slurred speech and time of onset. Another easy way to remember to signs of stroke is the 6S method or the BEFAST method.

#### Stroke: Remember the 6S Method to Diagnose Stroke

1. **S**udden (symptoms usually start suddenly)
2. **S**lurred Speech (speech is not clear, as if drunk)
3. **S**ide Weak (face, arm or leg or all three can get weak)
4. **S**pinning (vertigo)
5. **S**evere headache
6. **S**econds (note the time when the symptoms start and rush to the hospital)

Befast: **B**alance (loss of balance/dizziness), **E**yes (disturbance of vision in one or both eyes), **F**ace (facial droop), **A**rm (weakness)and **S**peech (slurred) **t**est.

All the symptoms need not be present to diagnose stroke. Any of the above mentioned symptoms can be present and is helpful is diagnosing ischemic as well as hemorrhagic stroke.

**Fig. 1:**
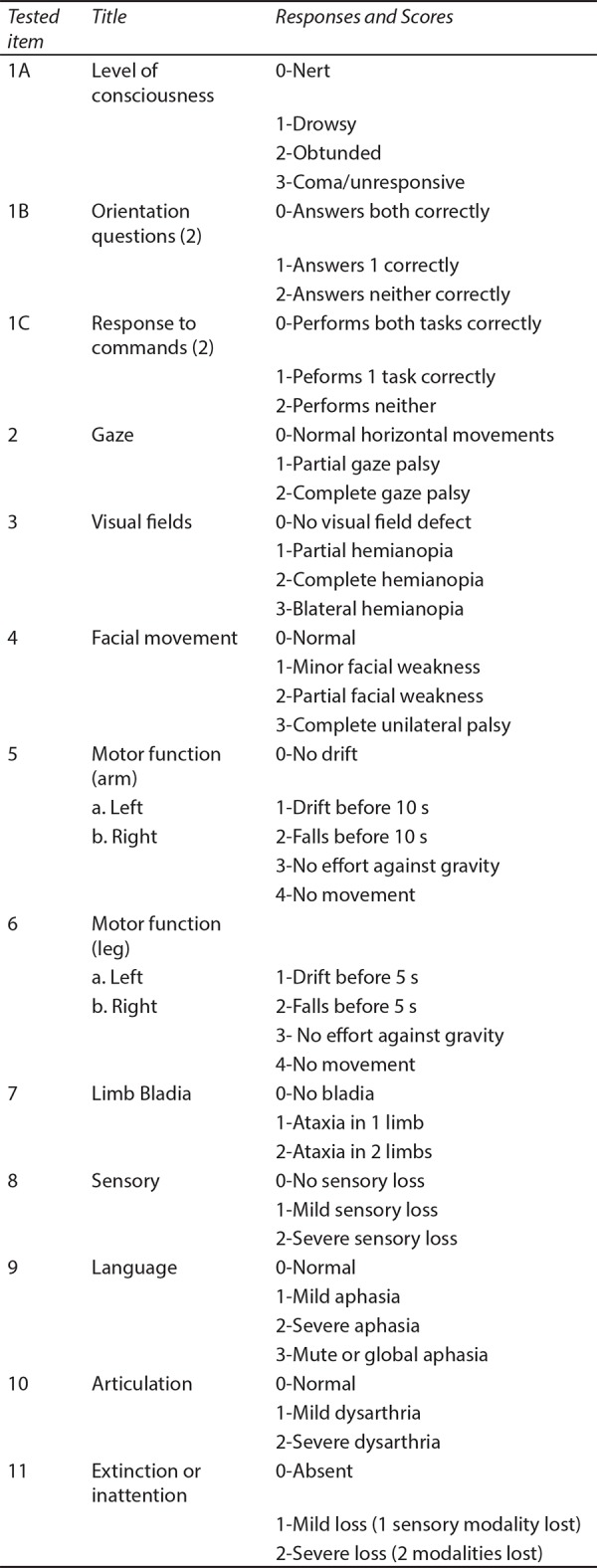
NIHS stroke scale

For complete assessment of a stroke patient it is recommended to use the NIH stroke scale (Fig. 1). According to the latest stroke metrics a patient with acute stroke should be examined by a trained physician or a neurologist within 10 minutes of arrival to the emergency room.

### Management of Acute Stroke

The most important factor in the management of acute ischemic stroke is time. The patient with ischemic stroke loses 190,0000 brain cells every minute, about 14000,000,000 nerve connections are destroyed every minute and 12 km (7.5 miles) of nerve fibres are lost every minute. The brain ages 3.6 years for every hour it is deprived of blood supply.^14^ There are two modalities of treatment available for treatment of acute ischemic stroke. Intravenous thrombolysis and mechanical thrombectomy.

Once the clinical diagnosis of an acute stroke is made the following steps need to be followed.

- Ensure that the patient is medically stable.
- Evaluate for reversible causes of neurological symptoms.
- Determine the nature of stroke (ischemic vs hemorrhagic)
- Treatment of stroke.
- Determine the causes of stroke (beyond the scope of this chapter).

#### Ensure the Patient is Medically Stable: Never Forget the ABC

In the treatment of acute stroke time is of the essence. Medical stability of the patient should be established as soon as possible so that stroke management can proceed. Airway, breathing and circulation need to be assessed like in every medical emergency. Large strokes, intracranial hemorrhage, strokes affecting posterior circulation present with loss of consciousness, bulbar dysfunction and sometimes respiratory distress. Hypoxia should be avoided at all costs and intubation should be considered if the airway is not protected or the patient needs ventilator support.

#### Evaluation of Reversible Causes of Neurological Symptoms

As per the stroke guidelines of finger stick glucose is an essential test before thrombolysis is started.^15^

Following tests can be considered in special patient groups, however none of them should delay the start of thrombolytic therapy if indicated unless the patient has a history of bleeding disorder, is taking anticoagulants or has history of thrombocytopenia:

- Electrocardiogram (this should not delay the noncontrast brain CT)
- Complete blood count including platelets
- Troponin
- Prothrombin time and international normalized ratio (INR)
- Activated partial thromboplastin time
- Ecarin clotting time, thrombin time, or appropriate direct factor Xa activity assay (if the patient is taking new oral anticoagulants)

Once the acute management of stroke is done other lab tests can be done such as liver function, kidney function etc.

#### Determine the Nature of Stroke (Ischemic vs Hemorrhagic)

Plain Head CT scan is sufficient to rule out intracranial hemorrhage. Systems should be established so that brain imaging studies can be performed within 20 minutes of arrival in the ED in at least 50% of patients who may be candidates for IV alteplase and/or mechanical thrombectomy (door to imaging time).^15^

Depending on the time of onset of symptoms further studies can be conducted to aid in management.^15^

- *If the patient presents within first 6 hours of symptoms onset:* CT scan should be combined with CT angiogram of the brain and the neck to rule out large vessel occlusion. CT angiogram should not be delayed to wait for serum creatinine. Whenever possible CT angiogram should be completed with the CT scan to save time for possible mechanical intervention.
  MRI or MR angiogram or MR perfusion is not indicated within the first 6 hours of symptom onset.
- *For patients presenting between 6 hours and 24 hours of symptom onset:* In selected patients with acute ischemic stroke within 6–24 hours of last known normal who have large vessel occlusion in the anterior circulation, obtaining CT perfusion, Diffusion weighted-MRI, or MRI perfusion is recommended to aid in patient selection for mechanical thrombectomy.

### Intravenous Thrombolysis

As recommended by AHA/ASA intravenous tPA infusion is treatment modality of choice for patients presenting within first 3 hours of onset of symptoms. The treatment window can be extended to 4.5 hours for eligible patients.^15,16^ Use of intravenous alteplase is based on the landmark National Institute of Neurological Disorders and Stroke (NINDS) tPA trial which showed that patients who received tPA were at least 30% more likely to have minimal or no disability at 3 months on the assessment scales. Outcome measures were in favour of the group that received tPA (global odds ratio for a favorable outcome, 1.7; 95 percent confidence interval, 1.2 to 2.6).^17^ A pooled analysis of ATLANTIS, ECASS, and NINDS was published in 2004 which showed that the tPA window could be extended to 4.5 hours with reasonable benefit to the patient.^18^ The European Cooperative Acute Stroke Study III (ECSASS-III) definitively established the efficacy of tPA within 3–4.5 hour window. ECASS-III showed that patients who received tpa within 3–4.5 hour window had a better chance to a favorable outcome with an odds ratio of 1.42 (95% CI 1.02-1.98, *P* = .04).^19^

Intravenous tPA infusion can only be given to eligible patients and there are strict inclusion and exclusion criteria as per stroke guidelines published in 2018.^15^

The checklist includes some FDA-approved indications and contraindications for administration of IV rtPA for acute ischemic stroke. Recent guideline revisions have modified the original FDA-approved indications. A physician with expertise in acute stroke care may modify this list.

Onset time is defined as either the witnessed onset of symptoms or the time last known normal if symptom onset was not witnessed.

In patients without recent use of oral anticoagulants or heparin, treatment with IV rtPA can be initiated before availability of coagulation test results but should be discontinued if INR is >1.7 or PT is abnormally elevated by local laboratory standards.

In patients without history of thrombocytopenia, treatment with IV rtPA can be initiated before availability of platelet count but should be discontinued if platelet count is <100,000/mm^3^.

aPTT indicates activated partial thromboplastin time; CT, computed tomography; ECT, ecarin clotting time;FDA, Food and Drug Administration; INR, international normalized ratio; IV, intravenous; PT, partial thromboplastin time; rtPA, recombinant tissue plasminogen activator; and TT, thrombin time.

There are additional relative exclusion criteria for patients in 3–4.5 hour window that determine if the patients is eligible for tPA or not.

A primary goal of achieving door to needle time (DTN) within 60 minutes in ≥50% of AIS patients treated with IV alteplase should be established. It may be reasonable to establish a secondary DTN time goal of achieving DTN times within 45 minutes in ≥50% of patients with AIS who were treated with IV alteplase.^15^

Dose of tPA: As per the guidelines infuse 0.9 mg/kg (maximum dose 90 mg) over 60 minutes, with 10% of the dose given as a bolus over 1 minute.

For treatment within 3 hours of stroke onset, alteplase led to a good outcome for 33% vs 23% for control (odds ratio [OR] 1.75, 95% CI 1.35-2.27). The number needed to treat (NNT) for one additional patient to achieve a good outcome was 10.^20.^

For treatment from 3 to 4.5 hours, the proportion with a good outcome in the alteplase and control groups was 35% and 30% respectively (OR 1.26, 95% CI 1.05–1.51, NNT 20).^20^

**Table 1:** Tpa inclusion and exclusion criteria

*Inclusion criteria*
Diagnosis of ischemic stroke causing measurable neurological deficit
Onset of symptoms <3 hours before beginning treatment
Aged ≥18 years
*Exclusion criteria*
Significant head trauma or prior stroke in previous 3 months
Symptoms suggest subarachnoid hemorrhage
Arterial puncture at noncompressible site in previous 7 days
History of previous intracranial hemorrhage
Intracranial neoplasm, arteriovenous malformation, or aneurysm
Recent intracranial or intraspinal surgery
Elevated blood pressure (systolic >185 mm Hg or diastolic >110 mm Hg)
Active internal bleeding
Acute bleeding diathesis, including but not limited to
Platelet count <100,000/mm^3^
Heparin received within 48 hours, resulting in abnormally elevated aPTT greater than the upper limit of normal
Current use of anticoagulant with INR >1.7 or PT >15 seconds
Current use of direct thrombin inhibitors or direct factor Xa inhibitors with elevated sensitive laboratory tests (such as aPTT, INR, platelet count, and ECT; TT; or appropriate factor Xa activity assays)
Blood glucose concentration <50 mg/dL (2.7 mmol/L)
CT demonstrates multilobar infarction (hypodensity >1/3 cerebral hemisphere)
*Relative exclusion criteria*
Recent experience suggests that under some circumstances—with careful consideration and weighting of risk to benefit—patients may receive fibrinolytic therapy despite 1 or more relative contraindications. Consider risk to benefit of IV rtPA administration carefully if any of these relative contraindications are present: Only minor or rapidly improving stroke symptoms (clearing spontaneously) Pregnancy Seizure at onset with postictal residual neurological impairments Major surgery or serious trauma within previous 14 days Recent gastrointestinal or urinary tract hemorrhage (within previous 21 days) Recent acute myocardial infarction (within previous 3 months)

**Table 2:** Special considerations for Tpa

*Aged >80 years* For patients >80 y of age presenting in the 3–4.5 hours window, IV alteplase is safe and can be as effective as in younger patients (New Recommendation)
*Severe stroke (NIHSS >25)* The benefit of IV alteplase between 3 hours and 4.5 hours from symptom onset for patients with very severe stroke symptoms (NIHSS > 25) is uncertain.† (Class IIb; LOE C-LD) (New recommendation)
*Taking an oral anticoagulant regardless of INR* For patients taking warfarin and with an INR ≤1.7 who present in the 3–4.5 hours window, IV alteplase appears safe and may be beneficial.† (Class IIb; LOE B-NR) (New recommendation)
*History of both diabetes and prior ischemic stroke* In patients with prior stroke and diabetes mellitus presenting in the 3–4.5 hours window, IV alteplase may be as effective as treatment in the 0–3 hours window and may be a reasonable option. (Class IIb; LOE B-NR) (New recommendation)

The benefit of intravenous (iv) defibrinogenating agents and of IV fibrinolytic agents other than alteplase and tenecteplase is unproven; therefore, their administration is not recommended outside a clinical trial. Tenecteplase administered as a 0.4 mg/kg single IV bolus has not been proven to be superior or noninferior to alteplase but might be considered as an alternative to alteplase in patients with minor neurological impairment and no major intracranial occlusion.^15^

### Endovascular Treatment for Stroke

Intravenous tPA has been the backbone of stroke treatment for almost 20 years. Intravenous therapy although effective has specific inclusion and exclusion criteria which limits its use in a large number of patients (Tables 1 and 2). Moreover the intravenous tPA cannot be used after the 4.5 hour window and has limited efficacy in patients with large vessel occlusion. Riedel et al showed that it is almost impossible to dissolve large clots with intravenous therapy.^21^ In another study by Alexandrov and Grotta up to one third of patients had re-occlusion of blood vessels after initial recanalization.^22.^

Over the last two decades the field of endovascular stroke intervention has grown exponentially. From initial trials of intra-arterial tPA infusion to initial clot retrieval devices and now to stent retriever technology the field has come a long way indeed. Initial trials, PROACT I and II showed promise with intra-arterial pro-urokinase infusion with improved outcomes and recanalization rates, however pro-urokinase was never approved by the FDA citing need for further confirmatory trials.^23,24^ Intra-arterial therapy theoretically sounds promising but has never been shown to improve outcomes or decrease mortality in randomised trials, thus is not the preferred approach today.

First generation device, the MERCI device, was approved for mechanical thrombectomy by the FDA and marked the beginning of era of endovascular stroke intervention. The MERCI device was tested in the MERCI and MULTI-MERCI trials both which had significantly high mortality (44 and 34% respectively), despite that the patients who had successful recanalization had a better chance at achieving good functional outcome.^25,26^ These trials laid the foundation for further research in the field of acute stroke intervention.^27^

**Fig. 2:**
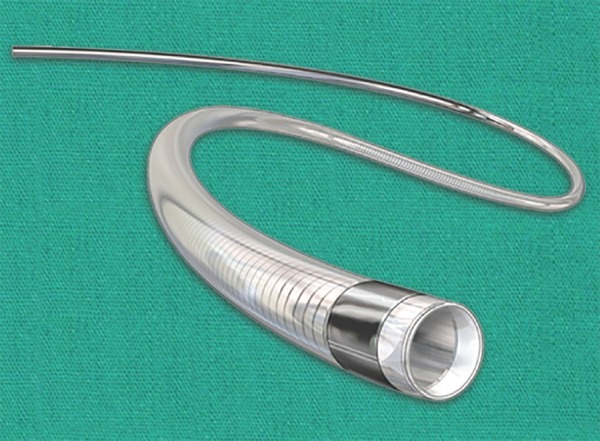
Aspiration catheter

**Fig. 3:**
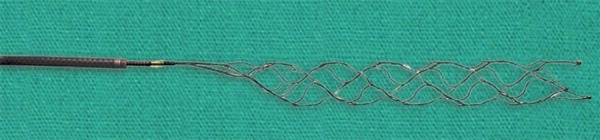
Stent retriever

In 2013, three landmark trials SYNTHESIS, MR RESCUE, and IMS III were published and disappointingly all three trials ruled against endovascular stroke intervention. The SYNTHESIS trial compared treatment with intravenous tPA alone to intra-arterial thrombolysis with rtPA, mechanical clot disruption or retrieval or a combination of these approaches. There was no difference in good clinical outcome (42%) in the endovascular arm vs (46%) in the intravenous tPA arm. Mechanical device was used in only 56/181 patients and stent retrievers were only used in 23 patients.^28^ MR RESCUE trial compared patients receiving standard care vs endovascular therapy using the MERCI device and the PENUMBRA system (Fig. 2). Patients with intracranial ICA or M1 occlusion, NIHSS greater or equal to 6 and within 8 hours of symptoms onset were enrolled. This trial showed no difference in outcomes of patients in the two groups. Reperfusion, defined as modified thrombolysis in cerebral infarction score (mTICI) 2a/3 was 67% and defined as mTICI 2b/3 was only 27%. Good clinical outcome (mRS 0–2) was only demonstrated in 14%.^28^ IMSIII trial showed a similar outcome with 41% good clinical outcome (mRS 0–2) in the combined Intravenous therapy (IVT)/Endovascular therapy arm vs 39% in the IVT only arm.^30^ IMS III did not require demonstration of vascular occlusion until the trial was midway when CT angiogram was included in the study protocol. IMS III also did not standardize the endovascular therapy as a result; the methods were a mix of pharmacological thrombolysis, manipulation of clot with use of a guidewire or microcatheter, mechanical and aspiration thrombectomy, and stent retriever technology (Figs 2 and 3).^30^ These trials failed to show any benefit of endovascular intervention over the intravenous therapy for a variety of reasons. These trials did not standardise the non invasive vascular imaging thus in IMS III more than 50% patients did not undergo vascular imaging. The endovascular therapy was performed using older devices and thus the results were often not promising. There were long delays from stroke onset to revascularization, in part due to lack of rapid workflow.^27^

Learning from the shortcomings of the trials done in 2013, multiple trials were started using standardized endovascular and imaging approaches. Six trials published from 2014 to 2016 showed statistically significant benefit of patients with anterior circulation strokes who presented within 6 hours of onset of symptoms.^31-36^ These trials have established the efficacy of mechanical thrombectomy over intravenous therapy with patients doing considerably well in the endovascular arm. The meta- analysis of the trials including the three negative trials IMSIII, SYNTHESIS and MR Rescue trial have shown benefit of endovascular intervention. The adjusted combined odds ratio for reduced disability at 90 days was 2.49, 95% CI 1.76–3.53, *p* < 0.0001 and number needed to treat (NNT) to reduce the disability by at least one point was 2.6. The largest meta-analysis to date included the above eight trials and also preliminary data from THERAPY and THRACE trial, the rate of functional independence (i.e, a 90-day modified Rankin scale [mRS] score of 0 to 2) was significantly greater for the intervention group compared with the control group (46 vs 27 percent, odds ratio [OR] 2.35, 95% CI 1.85-2.98).^37^

Recent success of DAWN and DEFUSE 3 trials has further extended the window for mechanical thrombectomy from 6 to 24 hours. These trials have also made it easier to manage patients presenting with wake-up strokes or unknown time of onset in the preceding 24 hours. DAWN trial enrolled 206 patients with stroke onset between 6 hours and 24 hours. There was a 33% adjusted difference in functional outcome in favour of the intervention arm. The number needed to treat for one additional patient to achieve functional independence was 3.^38^ Likewise in the DEFUSE 3 trial there was a 28% adjusted difference in functional outcome in favour of the intervention arm and therefore the number needed to treat for one additional patient to achieve functional independence was 3.6.^39^

### Complications of Intravenous Thrombolysis

Complications related to intravenous r-tPA include symptomatic intracranial hemorrhage, major systemic hemorrhage, and angioedema in approximately 6%, 2%, and 5% of patients, respectively. Guidelines recommend strict blood pressure control and serial neurological exams to diagnose intracranial hemorrhage.^15,40^ In the event that an intracranial hemorrhage is suspected it is recommended to stop alteplase infusion and perform stat labs which include a CBC, PT (INR), aPTT, fibrinogen level, and type and cross-match. An emergent nonenhanced head CT should be performed to evaluate further. Intravenous cryoprecipitate (includes factor VIII), at a dose of 10 U infused over 10–30 minutes (onset in 1 hour, peaks in 12 hours) should be administered and may be repeated for fibrinogen level of <200 mg/dL. Tranexamic acid or ε-aminocaproic acid may also be used. Stat hematology and neurosurgery consultations should be sought. Supportive therapy should continue.^15^ Angioedema should be treated like an allergic reaction and emergent intubation may be needed it breathing difficulty is present. Use antihistamines and steroids are recommended in managing angioedema.^15^

## CONCLUSION

Management of acute ischemic stroke is time dependent. Efficient and effective stroke care depends on a well functioning team from the emergency room to the neurologist and the interventional neurologist. Accurate diagnosis, emergent management to stabilize the patient and correct choice of imaging can make a lot of difference in the outcome of a patient. Every minute lost in wrong imaging or lab test results in decrease in functional outcome and ultimately irreversible paralysis. Success of stroke treatment is dependent on the entire team working smoothly and efficiently. As the times evolve, more and more centres will come up with dedicated stroke teams just like cardiologists manage acute myocardial infarction. Although future of stroke care is bright, two major roadblocks persist public awareness and hospital competence. Hopefully, this chapter will help with at least one of them.
